# New Surgical Approach and Rehabilitation After Cemented Total Ankle Arthroplasty

**DOI:** 10.7759/cureus.75398

**Published:** 2024-12-09

**Authors:** Gensuke Okamura, Makoto Hirao, Takaaki Noguchi, Yuki Etani, Kosuke Ebina, Hideki Tsuboi, Seiji Okada, Jun Hashimoto

**Affiliations:** 1 Orthopaedic Surgery, National Hospital Organization, Osaka Minami Medical Center, Kawachinagano, JPN; 2 Orthopaedic Surgery, Graduate School of Medicine, Osaka University, Suita, JPN; 3 Musculoskeletal Regenerative Medicine, Graduate School of Medicine, Osaka University, Suita, JPN; 4 Orthopaedic Surgery/Rheumatology, Osaka Rosai Hospital, Sakai, JPN

**Keywords:** early full-weight bearing exercise, early range of motion (rom) exercise, early rehabilitation program, total ankle arthroplasty (taa), wound healing

## Abstract

Background: According to the conventional postoperative procedure after total ankle arthroplasty (TAA) for end-stage osteoarthritis (OA) and rheumatoid arthritis (RA), mobilization and weight-bearing are currently started after completion of wound healing. Recently, an early rehabilitation program after cemented TAA with a modified anterolateral approach has been attempted because this approach could provide stable wound healing. To investigate the possibility of expediting rehabilitation, this study evaluated the feasibility, safety, and universality of an early rehabilitation program after cemented TAA using a modified anterolateral approach, even when a surgeon was completely changed.

Methods: This retrospective, observational study investigated 13 consecutive ankles (OA: 11 ankles, RA: two ankles) that had undergone cemented TAA with a modified anterolateral approach. As an early rehabilitation program, after early dorsiflexion mobilization (day three), full weight-bearing/gait exercise was started seven days after surgery (10 days after if malleolar osteotomy was added). Postoperative wound complications were observed and recorded. The number of days of hospitalization was also evaluated. Range of motion (ROM) of dorsiflexion/plantarflexion was measured. Patients also completed the self-administered foot evaluation questionnaire (SAFE-Q) and the Japanese Society for Surgery of the Foot (JSSF) ankle/hindfoot score preoperatively and at the final follow-up.

Results: No postoperative complications related to wound healing were observed even after the early rehabilitation program. The duration of hospitalization was shorter (23.5 days) than our previous experience after a conventional rehabilitation program (36-38 days). ROM for both dorsiflexion (from 4.6° to 16.5°; p=0.002) and plantarflexion (from 27.7° to 37.7°; p=0.019) increased significantly, and all indices of the SAFE-Q score and the JSSF score showed highly significant improvement.

Conclusions: An early rehabilitation program was feasible and safe following the modified anterolateral approach. Although these points were confirmed with a cemented TAA system at present, further innovations in postoperative rehabilitation after TAA are expected.

## Introduction

Various treatment options are available for end-stage ankle osteoarthritis (OA), including conservative therapy, supramalleolar osteotomy, ankle joint arthrodesis, and total ankle arthroplasty (TAA). In these last four to five decades, as an effective alternative to arthrodesis, TAA has been performed for end-stage ankle OA with the significant benefits of pain relief, preservation of joint range of motion (ROM), and improvement of function [1,2].

Although there have been some modifications of implant design and surgical procedures, as mentioned above, there has been little progress in peri-postoperative rehabilitation programs for patients who have undergone TAA in these last decades. Several postoperative guidelines for rehabilitation have been applied [3-5], but none of the present guidelines has described mobilization from the early phase after surgery. Ankle mobilization according to existing protocols is currently started at least two to three weeks postoperatively. Resting, immobilization, and non-weight-bearing have been recommended for zero to two weeks after surgery to allow wound healing. During this period, mobilization is allowed only for the toes. These protocols for mobilization and weight-bearing after TAA have not been changed for many years. However, early (about 24 hours after surgery) knee-bending exercise has increasingly been adopted after TKA [6]. When performing rehabilitation after TAA, the duration of immobilization and non-weight-bearing for the affected ankle should correspond to the situation, including differences between approaches such as anterior and lateral approaches, and differences between cemented and non-cemented implants. In our group, a system with an anterior approach and cemented fixation has been used for TAA, and then, in the present study, progress in rehabilitation with such a system for TAA is described. The difference in early-phase postoperative procedures between TKA and TAA is due to the prioritization of wound healing. Mobilization is allowed after completion of wound healing following TAA because mobilization causes tension on the skin and subcutaneous tissues, thus potentially delaying and/or causing failure of wound healing. In the conventional anterior approach, the skin incision is placed directly above the tibialis anterior (TA)/extensor hallucis longus (EHL) tendon. In the process of wound healing, once the retinaculum of the compartment for the TA tendon is opened under mobilization-induced tension, the bowstring phenomenon (pushing up the skin and/or retinaculum by the TA tendon) can seriously delay wound healing [7]. Furthermore, tenomodulin, a protein secreted from the tendon, is known to inhibit angiogenesis and is also thought to delay wound healing by interfering with the vascularization of dermal tissues directly overlying the exposed TA tendon [8]. A modified anterolateral approach has recently been introduced in which the skin incision is not placed directly above the TA/EHL tendon, and the retinaculum around the TA tendon is not opened [9]. Using this approach allows stable wound healing and thus reduces the number of days until suture removal. Taken together with such stability of wound healing, early mobilization and early full weight-bearing after TAA were considered feasible with the modified anterolateral approach. In recent years, we first tried the protocol for early dorsiflexion ROM exercise after cemented TAA because the bowstring phenomenon induced by TA tendon could occur, especially with plantarflexion of the ankle joint, and the safety of such an early ROM exercise protocol was confirmed [10]. In the next step, we tried not only the early ROM exercise protocol, but also the protocol for early full weight-bearing and walking, and then we could also confirm the safety and its usefulness [11]. To investigate the possibility of expediting rehabilitation, this study evaluated the feasibility and safety and the universality of an early rehabilitation program after cemented TAA using a modified anterolateral approach, even when a surgeon was changed.

## Materials and methods

This retrospective, observational study investigated 13 consecutive patients with 13 ankles (osteoarthritis: 11 ankles, rheumatoid arthritis (RA): two ankles) in a single institution that had used a mobile-bearing ankle prosthesis (FINE mobile-bearing prosthesis; Teijin-Nakashima Medical Co., Okayama, Japan) [12-14] for treatment of end-stage ankle deformity/destruction from April 2023 to February 2024. The subject was a completely different series from our previous studies [10,11], and the surgery was performed by a single surgeon: (G.O) who was completely different from the surgeon in previous studies [10,11]. The demographic characteristics of the patients are shown in Table 1. This research was performed in compliance with the Declaration of Helsinki and was approved by the institutional review boards of the authors’ affiliated institutions. Written, informed consent was obtained from all patients.

**Table 1:** Preoperative characteristics of patients Data are presented as mean±SD unless otherwise noted. BMI: body mass index; OA: osteoarthritis; TCZ: tocilizumab; JAKi: Janus kinase inhibitor; DAS28-CRP score: disease activity score of 28 joints; C-reactive protein

Age (years)	74.5±8.8
Height (cm)	152.7±7.3
Body weight (kg)	55.5±10.8
BMI (kg/m^2^)	23.7±3.8
Male:female (n)	2:11
RA:OA	2:11
RA disease duration (years)	16.5±8.5
Steinbrocker classification	
Stage (No. of ankles)	III (0) IV (2)
Functional class (No. of ankles)	I (1) II (1)
Preoperative DAS28-CRP score	2.59±0.23
Prednisolone usage (%)	0
Prednisolone dosage (mg/day)	0
Methotrexate usage (%)	50
Methotrexate dosage (mg/week)	8.0±0.0
Biologics usage (%)	50
Biologics or JAKi used (No. of ankles)	TCZ (1), JAKi (1)

Surgical technique

Modified Anterolateral Approach

The modified anterolateral approach was performed as described previously [9-11]. The skin incision was based on the conventional anterolateral approach/Bohler’s incision [15]. The incision was made one finger-width (1.5-2.0 cm) laterally from the EHL, and then the superficial peroneal nerve was identified and retracted. The medial side of the stem of the inferior extensor retinaculum was opened without intervention to the superior and inferior subdivisions of the superomedial band of the inferior extensor retinaculum, which forms a tunnel for the TA tendon [9]. The extensor digitorum longus (EDL) was then retracted laterally, and the TA/EHL compartment was retracted medially. The approach to fat tissue was continued, and then the dorsalis pedis artery/vein and deep peroneal nerve were retracted medially or laterally. If these neurovascular bundles shifted laterally, lateral retraction would be easy for the approach, but if the branch artery to the medial malleolus could be preserved, medial retraction of the bundle would be recommended to maintain the blood supply to the medial malleolus and talus. The exposed superficial peroneal nerve was gently retracted [9].

TAA and postoperative procedures

Surgery was performed as described previously using a mobile-bearing ankle prosthesis design (FINE mobile-bearing prosthesis; Teijin-Nakashima Medical Co.) [12-14]. After preparation for tibia/talus implantation, medial malleolar lengthening osteotomy (preserving the periosteum) was added if the tightness of soft tissues was not acceptable [14,16]. Both tibial and talar components were implanted with bone cement. Routine wound closure was completed with careful suturing of the ankle joint capsule and extensor retinaculum. The subcutaneous layer was sutured as much as possible with 3-0 absorbable thread. The skin layer was sutured with 4-0 nylon vertical mattress sutures. Intraoperative events and/or optional procedures are described in Table 2. The ankle was placed gently under the support of soft splint fixation. Patients were prohibited from dorsiflexion/plantarflexion within three days after surgery, but calf massages and toe motion exercises were started one day after surgery. After three postoperative days, both active and passive range of motion (ROM) exercises for dorsiflexion were started without weight-bearing. Full weight-bearing and gait exercises were started seven days after surgery (10 days after if malleolar osteotomy was added) [11].

**Table 2:** Intraoperative events Tourniquet time and operation time are given as the mean±standard deviation.

Medial malleolar osteotomy (%)	46.2 (6 of 13)
Lateral malleolar osteotomy (%)	0 (0 of 13)
Medial malleolar fracture (%)	0 (0 of 13)
Lateral malleolar fracture (%)	0 (0 of 13)
Gastrocnemius recession (%)	61.5 (8 of 13)
Concomitant subtalar joint/ talonavicular joint fusion (%)	0 (0 of 13)
Tourniquet time (min)	113.3±40.2
Operation time (min)	174.2±35.8

The duration of soft splint fixation was not changed if malleolar osteotomy was added, as in malleolar fracture cases. However, when patients were allowed full-weight-bearing, it was with an attached ankle brace for eight weeks to avoid ankle sprains [14]. At the same time, gait exercises were also started.

Clinical assessments

The number of days to suture removal after TAA was recorded. Suture removal after TAA was planned for around two weeks after TAA with careful observation of the wound state. Occurrences of postoperative events, such as blister formation, eschar formation (width >10 mm) on the wound, and wound dehiscence, were observed and recorded. The decreased peri-incisional sensation was checked since the superficial peroneal nerve had been retracted intraoperatively. These investigations were based on the evaluation previously described [9-11]. As a criterion for suture removal, confirmation that there was no exudate from the wound and no wound dehiscence even if tension to both sides of the wound was applied was required.

The ROM of dorsiflexion/plantarflexion was also measured preoperatively and at the final follow-up. ROM measurement was based on the methods in the previous reports [17,18]. The length of hospitalization was also counted as days. Because there are differences in the instructions/guidelines for the length of hospitalization between hospitals, ankles in a single institution were evaluated in this study. As a criterion for hospital discharge, the patient should be able to walk on flat ground alone or with a T-cane and be able to go up and down stairs alone or hold a handrail.

For cases of RA, disease activity was assessed using the disease activity score of 28 joints and the C-reactive protein score [19].

As clinical assessments, patients completed a self-administered foot evaluation questionnaire (SAFE-Q) [20] and the Japanese Society for Surgery of the Foot (JSSF) ankle/hindfoot score (based on the American Orthopaedic Foot and Ankle Society score with modifications for the Japanese population) [21,22] preoperatively and at the final follow-up.

Statistical analysis

Differences between preoperative and postoperative data were assessed by a paired Wilcoxon signed-rank test. Significance was set at p < 0.05. Statistical analyses were performed using EZR (Saitama Medical Center, Jichi Medical University, Saitama, Japan) [23].

## Results

Number of days to suture removal

The mean number of days to suture removal was 12.8 days. After removal, no occurrence of postoperative events was seen even after early mobilization for dorsiflexion, early full weight-bearing, and gait exercise (Table 3).

**Table 3:** Postoperative status of the wound site and days of suture removal and hospitalization

Blister formation (%)	15.4 (2 of 13)
Eschar formation (width >10mm) (%)	15.4 (2 of 13)
Wound dehiscence (%)	0 (0 of 13)
Exposure of TA tendon (%)	0 (0 of 13)
Deep infection (%)	0 (0 of 13)
Peri-incisional decreased sensation (%)	15.4 (2 of 13)
Days to suture removal (days)	12.8±1.1
Days of hospitalization (days)	23.5±4.4

Length of hospitalization

The mean length of hospitalization was 23.5 days (Table 3).

Ankle ROM

ROM for both dorsiflexion and plantarflexion showed significant improvement after surgery (dorsiflexion: from 5° to 17° and plantarflexion: from 28° to 38°) (Table 4).

**Table 4:** Comparison of ankle motion and clinical scores between preoperatively and at the final follow-up Values are given as the mean and standard deviation. Differences between preoperative and postoperative data were assessed by a paired Wilcoxon signed-rank test. A p-value of <0.05 was statistically significant.

	Preoperative	Final follow-up	P-value
Range of ankle motion			
Dorsiflexion (degrees)	4.6±6.0	16.5±3.0	0.002
Plantar flexion (degrees)	27.7±13.8	37.7±6.9	0.019
Arc of motion (degrees)	32.3±14.9	54.2±7.1	0.0016
SAFE-Q scores			
Pain and pain-related (100)	43.5±17.2	85.0±8.5	0.00024
Physical functioning and daily living (100)	34.3±17.6	73.4±5.8	0.0017
Social functioning (100)	41.0±22.9	81.4±8.1	0.0017
General health and well-being (100)	41.9±12.0	79.6±5.0	0.0016
Shoe-related (100)	57.1±16.3	88.5±4.1	0.0016
JSSF ankle/hindfoot scales			
Pain (40)	15.4±8.4	39.2±2.7	0.0011
Function (50)	22.5±10.2	39.1±5.4	0.0011
Alignment (10)	6.2±2.9	10.0±0.0	0.0048

Clinical assessments

Significant improvements in both the SAFE-Q and JSSF ankle/hindfoot scores were seen after surgery (Table 4).

## Discussion

**Figure 1:**
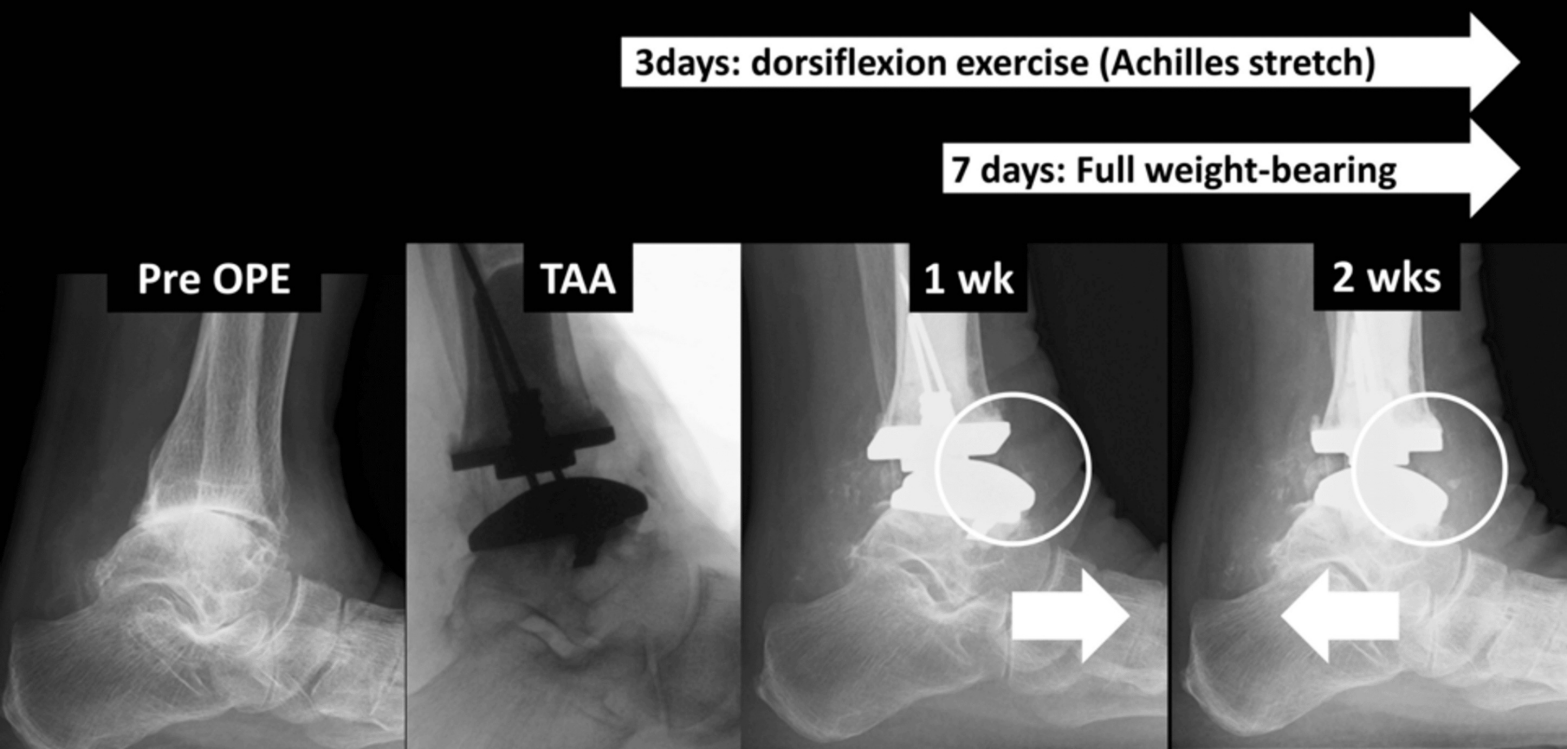
Transition of talar bone location during the perioperartive period in lateral view radiographs at weight-bearing Radiograph images belong to one of the patients in the current study. After TAA, the anterior shift of the talar bone remained for one week after surgery. However, Achilles tendon stretch by passive/active dorsiflexion three days after surgery and full weight-bearing/walking seven days after surgery contributed to relocation to the posterior side of the talar bone. This effect could contribute to the natural movement of the ankle joint.

This study reconfirmed that an early rehabilitation program (early mobilization, weight-bearing, and gait exercise) after cemented TAA using a modified anterolateral approach, which we reported before [10,11], was feasible and safe. At the same time, the early rehabilitation program appeared to contribute to shortening the length of hospitalization (23.5 days) compared with the conventional program (both mobilization and weight-bearing were started about two to three weeks after surgery), which we reported previously (37.6 days) [11]. Based on these facts, these surgical approaches and rehabilitation protocols were considered to be stable and universal even if the surgeon was changed completely. From the perspective of the generous medical insurance and social costs in the country, shortening hospital stays after orthopedic surgery is desirable. Furthermore, it is well known that hospitalization affects physical function and mobility, especially for older people [24], so hospitalization should be shortened as much as possible, especially for elderly patients. In the first place, TAA is often indicated for elderly patients. To discharge them from hospitals early after surgery, early acquisition of full weight-bearing and walking ability is desirable. A few reports allowed partial weight-bearing one week after surgery without ankle mobilization [25-28]. Rushing et al. reported the short-term outcomes of the INFINITY total ankle prosthesis with an early partial weight-bearing program, but 8 weeks were required to start full weight-bearing without any orthotics [28]. In the present series, full weight-bearing from one week after surgery was feasible and safe, suggesting significant progress in the weight-bearing program. It is also important to focus on full weight-bearing and gait exercise from the early phase in terms of preventing sarcopenia, osteopenia, disuse muscle atrophy, weakness, and subsequent loss of physical mobility due to prolonged bed rest [29-37]. Focusing on dorsiflexion mobilization early after TAA is important in terms of improving foot/ankle function and kinematics [38-44] and preventing deep vein thrombosis (DVT) [45,46]. Therefore, ROM exercise from the early period after TAA is also desirable. As shown in Figure 1, Achilles tendon stretching by early ROM exercise and full weight-bearing could contribute to the relocation of the talar bone, suggesting obtaining natural movement of the ankle joint.

Implementing early mobilization/weight-bearing after TAA also requires consideration of the initial fixation of the implants. Once tibial and talar components have been implanted with bone cement, primary fixation should be sufficiently rigid. The mechanics of the bone-implant boundary thus do not appear to be affected by when mobilization is started. This point might be a strong point of the cemented TAA system. However, whether early mobilization could be started even in cases of non-cemented TAA remains unclear. Since a biological interface between bone and the implant surface is required for non-cemented TAA, early mobilization during the period before primary fixation is sufficient might adversely affect bone-implant fixation. To start rehabilitation (ROM exercise and weight-bearing) after cemented TAA to obtain the merits mentioned above, the first priority is to achieve stable wound healing. With regard to wound healing, the surgical site was under conditions of coagulation and the inflammation phase of the wound-healing process within three days after surgery [47]. Thus, to prevent a high-pressure state in the subcutaneous compartment during the first three days after surgery, rest (immobilization), ice, compression, and elevation (RICE) treatment was prioritized over mobilization. Furthermore, a modified anterolateral approach, which we previously reported, allows stable wound healing and thus reduces the number of days to suture removal [9]. Indeed, no discharge from the wound was seen throughout the period after resuming mobilization and weight-bearing, and no wound dehiscence was seen after suture removal in the present study (Table 3). Based on these results, even if wound healing has not been completed, early mobilization for dorsiflexion, full weight-bearing, and gait exercise is possible and safe as long as a modified anterolateral approach is used for TAA.

Some limitations to this study need to be kept in mind. First, plantarflexion exercise was not allowed from the early phase of this study. This is because anterior soft tissues, including the anterior capsule, extensor retinaculum, and skin, are stretched during plantarflexion of the ankle joint (bowstring phenomenon) [7], and plantarflexion exercises were deliberately not introduced to avoid wound dehiscence. The timing of the plantarflexion exercise should be further discussed. Second, in cases of non-cemented TAA, the safety of early mobilization and gait exercise remains unclear. Since a biological interface between bone and the implant surface is required for non-cemented TAA, early mobilization during the period before adequate primary fixation is achieved might adversely affect bone implant fixation. Third, aiming to further expedite rehabilitation, earlier full weight-bearing and gait exercises need to be discussed. It has been previously reported that, with only five days of disuse, substantial skeletal muscle loss occurs [48]. Therefore, it is considered desirable to start full weight-bearing and gait exercises earlier than seven days after surgery, as in the present study. Fourth, although the number of days to suture removal and length of hospitalization was based on certain criteria described in the manuscript, these judgments were not performed by a single surgeon. These points are also limitations of this study.

## Conclusions

Even if the surgeon changes completely, an early rehabilitation program (early mobilization from three days, and early full weight-bearing and gait exercise from seven days after cemented TAA) was safe and feasible with the modified anterolateral approach. Further innovations in postoperative rehabilitation after TAA are expected.
